# PolyHaplotyper: haplotyping in polyploids based on bi-allelic marker dosage data

**DOI:** 10.1186/s12859-022-04989-0

**Published:** 2022-10-23

**Authors:** Roeland E. Voorrips, Giorgio Tumino

**Affiliations:** Wageningen University and Research – Plant Breeding, Wageningen, The Netherlands

**Keywords:** Haplotype, SNP array, Multi-allelic marker, Polyploid

## Abstract

**Background:**

For genetic analyses, multi-allelic markers have an advantage over bi-allelic markers like SNPs (single nucleotide polymorphisms) in that they carry more information about the genetic constitution of individuals. This is especially the case in polyploids, where individuals carry more than two alleles at each locus. Haploblocks are multi-allelic markers that can be derived by phasing sets of closely-linked SNP markers. Phased haploblocks, similarly to other multi-allelic markers, will therefore be advantageous in genetic tasks like linkage mapping, QTL mapping and genome-wide association studies.

**Results:**

We present a new method to reconstruct haplotypes from SNP dosages derived from genotyping arrays, which is applicable to polyploids. This method is implemented in the software package PolyHaplotyper. In contrast to existing packages for polyploids it makes use of full-sib families among the samples to guide the haplotyping process. We show that in this situation it is much more accurate than other available software, using experimental hexaploid data and simulated tetraploid data.

**Conclusions:**

Our method and the software package PolyHaplotyper in which it is implemented extend the available tools for haplotyping in polyploids. They perform especially well in situations where one or more full-sib families are present.

**Supplementary Information:**

The online version contains supplementary material available at 10.1186/s12859-022-04989-0.

## Background

Over the last two decades, tools for genetic analysis of polyploid species (species with more than the usual two complements of chromosomes) have been developed, including tools for linkage mapping, allele dosage scoring, haplotyping and QTL mapping (reviewed by [1]). Among those tools, some address the problem of identifying short-range haplotypes in populations and identifying the haplotype compositions of individuals in populations, together known as haplotyping. If we consider a set of tightly linked bi-allelic markers (e.g. single-nucleotide polymorphisms, SNPs with only two alleles in the population studied) as one genetic locus, then the haplotypes covering these markers can be interpreted as the alleles of that locus. Such a locus, which we will call a haploblock, can then be considered a multi-allelic marker locus, because generally more than two haplotypes may occur (see Additional file 1: Haploblocks and haplotyping.pdf). Recombination within such a haploblock should be very rare over the population studied. This can be achieved by selecting SNP markers known to be located on the same contig or very closely spaced according to a physical or genetic linkage map; the more diverse the population, the closer the SNPs in haploblocks should be spaced. Due to varying SNP density the number of SNPs available to create haploblocks can vary. In diploids, but even more so in polyploids, multi-allelic markers are more informative than bi-allelic markers. Especially for quantitative trait locus (QTL) detection through linkage mapping or genome-wide associating studies (GWAS) and for subsequent selection of specific QTL genotypes, one-to-one associations between marker alleles and QTL alleles are very informative, and these associations are far more likely with multi-allelic markers such as haploblocks than with bi-allelic markers such as SNPs. Even without such one-to-one associations multi-allelic markers will provide increased power of detection over the underlying single SNPs. Associations between multi-allelic markers and QTL alleles are also more likely to be valid in wider germplasm than that in which the association was established. Also linkage mapping might benefit from the additional information provided by haploblocks compared to single SNPs. Other types of multi-allelic markers have been used for these purposes, especially Short-Sequence Repeats (SSRs or microsatellites; [2]), but their application is limited by the low throughput of SSR assays and the limited number of SSRs available compared to SNPs.

Several short-range SNP phasing methods have been proposed for polyploids and implemented in software. Most of these are based on sequence reads, including HapCompass [3, 4], HAPLOSWEEP [5], Tripoly [6] and PopPoly [7], and Poly-Harch [8]. Here in contrast we focus on haplotyping based on SNP allele dosages derived from SNP arrays. Such methods have been published for diploids, e.g. HaploView [9], Beagle [10], AlphaImpute [11] and PedrPoly [12]. In polyploids the problem is considerably more complex. Some methods have been implemented in software packages including SATlotyper [13], polyHap [14, 15] SHEsisPlus [16–18] and Happy-inf [19]. These packages however do not use known pedigree relations among the individuals and therefore often return sub-optimal results. Our work aims to fill this gap with a method that uses any full-sib (FS) families present in the data to guide the imputation of haplotypes.

SNP allele dosages obtained from arrays are quite accurate, but they are not error-free and may include missing data; therefore an approach based on dosages must be able to deal with these problems.

Here we describe a method, implemented in R package PolyHaplotyper, and evaluate its results with simulated and real data from data sets with different pedigree structures. The examples were chosen to represent the situations for which PolyHaplotyper was designed, i.e. polyploid populations that include full-sib families. We also compare its performance to that of other software for performing haplotyping from unphased SNP dosage data.

## Results

### Data set 1: hexaploid Chrysanthemum population

Since the true haplotype compositions in this Chrysanthemum population were not known, we focused on the number of haplotyped individuals and the frequency of non-matching parent and offspring genotypes to evaluate the results of PolyHaplotyper.

In Table 1 we present the results separately for the 26 haploblocks with 4 SNP markers and the 17 haploblocks with 5 SNP markers, and for the 3 categories of individuals treated differently by PolyHaplotyper (FS individuals, FS parents and other individuals).

**Table 1:** PolyHaplotyper results with Data set 1

	SNP markers per haploblock		
	4	5	All
Nr of haploblocks	26	17	43
Mean nr of haplotypes	5.6	5.9	5.7
Fullsib individuals (571)			
Mean % fully genotyped	77.2	74.1	76.0
Mean % haplotyped	75.9	80.0	77.6
Fullsib parents (7)			
Mean % fully genotyped	93.4	91.6	92.7
Mean % haplotyped	79.7	84.9	81.7
Other individuals (53)			
Mean % fully genotyped	58.1	58.4	58.2
Mean % haplotyped	41.0	52.1	45.4
All individuals (631)			
Mean % fully genotyped	75.8	72.9	74.7
Mean % haplotyped	73.0	77.7	74.9
Mean % checkable	69.0	73.1	70.6
Mean % matching parent(s) (of all checkable)	99.9	100.0	99.9
Total nr matching parent(s)	11,306	7845	19,151
Total nr conflicting with parents	9	1	10
Total nr non-checkable	5091	2881	7972

The percentage of fully genotyped individuals (individuals with no missing SNP dosages in the haploblock) was larger with 4 than with 5 SNPs per haploblock, as expected. In contrast, the percentage of haplotyped individuals was larger in the haploblocks with 5 SNP markers, probably because there were less cases where two solutions were equally likely, and therefore also the number of individuals whose inferred haplotype could be checked against that of their parent(s) was larger. Almost no parent–offspring conflicts were present.

Some individuals were not haplotyped even if they had no missing SNP dosages, because multiple haplotype combinations were allowed by the SNP data and the other constraints (Mendelian inheritance, parsimonious haplotype sets). Among FS individuals also the reverse occurred: individuals were haplotyped while some SNP dosages were missing. In these cases the non-missing SNP genotypes allowed only one haplotype combination that was compatible with the inferred parental genotypes.

The number of haplotypes estimated to be present was slightly higher in haploblocks of 5 than of 4 markers, but not as much as might be expected from the theoretically possible numbers of haplotypes (16 for 4 markers, 32 for 5 markers).

The highest percentage of fully genotyped individuals was found among the parents. This is due to the fact that the parents were genotyped in multiple replicates, while all other individuals were genotyped only once; by merging the SNP dosage data of the replicates a consensus genotype with less missing data was obtained. The FS individuals were more often fully genotyped than the other (non-parent, non-FS) individuals, probably because the SNP array was developed using sequencing data from 5 of the 7 FS parents and some other genotypes [20]. The percentage of haplotyped individuals was highest among the FS parents, lower among the FS individuals and lowest among the other material. In all cases, the haplotyping results agreed with the observed marker dosages.

In order to assess the importance of using the information about the FS family structure we also haplotyped the individuals as if they were unrelated, but used the pedigree information to check for parent–offspring conflicts. Over all 43 haploblocks, without FS information 46.7% of the individuals were haplotyped, versus 74.9% with FS information, and 40.8% (versus 70.6%) could be checked. Of the checkable individuals 99.1% (versus 99.9%) matched with their parents.

We analyzed the same data set with two other haplotyping packages: SATlotyper [13] and Happy-inf [19] (Table 2). The results of Happy-inf vary somewhat between runs with the same input data, but not much; in Table 2 we show the results of a typical run. The results of SATlotyper, like these of PolyHaplotyper, do not vary. Because these packages do not use FS families or other substructure in the population, we present the results for all individuals together.

**Table 2:** Haplotyping results of SATlotyper and Happy-inf with Data set 1

	SATlotyper			Happy-inf		
SNP markers per haploblock	4	5	All	4	5	All
Nr of haploblock	26	17	43	26	17	43
Mean nr of haplotypes	5.4	6.4	5.8	8.0	9.9	8.7
Nr of individuals	631	631	631	631	631	631
mean % fully genotyped	75.8	72.9	74.7	75.8	72.9	74.7
mean % haplotyped	100.0	100.0	100.0	75.8	72.9	74.7
Mean % checkable	93.7	93.7	93.7	71.3	68.6	70.2
Mean % matching parent(s) (of all checkable)	81.5	81.9	81.7	91.6	91.9	91.7
Total nr matching parent(s)	12,525	8231	20,756	10,712	6734	17,446
Total nr conflicting with parents	2841	1816	4657	984	628	1612
Total nr non-checkable	1040	680	1720	4710	3365	8075

In contrast to PolyHaplotyper, SATlotyper haplotypes all individuals and Happy-inf haplotypes all fully genotyped individuals. However, in both cases the percentage matching their parents (81.7% and 91.7%) is much lower than that obtained by PolyHaplotyper (99.9%). In absolute terms the numbers of individuals matching their parents are not very different (19,151, 20,756 and 17,446 for PolyHaplotyper, SATlotyper and Happy-inf, respectively) but the number of individuals conflicting with their parents is much lower for PolyHaplotyper (10) than for SATlotyper (4657) and Happy-inf (1612). The computation time was 22 min for SATlotyper and 59 min for Happy-inf, versus 2 min for PolyHaplotyper (and 2.3 s for PolyHaplotyper without specified FS families).

The total number of haplotyped individuals matching their parents is higher for SATlotyper (20,756) and lower for Happy-inf (17,446), compared to that for PolyHaplotyper (19,151). The total number of conflicting haplotyping results is much higher for both packages (4657 and 1612) than for PolyHaplotyper (10).

Like for PolyHaplotyper, the haplotyping results of both these packages matched with the observed marker dosages, although in one haploblock 3 individuals without any marker dosages were also haplotyped by SATlotyper, with different haplotype combinations.

We also compared the PolyHaplotyper results with those of ShesisPlus [17, 18], which is accessible through a web interface [21]. Using this web interface is cumbersome, as it requires to enter the parameters and data, and to download the results, for each haploblock separately. Like Happy-inf, ShesisPlus is not deterministic: with the same input it generates different outputs. For that reason, we limited our use of ShesisPlus to two haploblocks with 4, and two haploblocks with 5 SNP markers, submitted each haploblock 3 times, and compared the results with those of PolyHaplotyper (Additional file 2: Table S1) as these appeared to be quite reliable, based on the high level of parent–offspring matching (100%, 100%, 99.8% and 100% for haploblocks ctg001, ctg002, ctg003 and ctg004, respectively). ShesisPlus does not produce haplotyping results for individuals but only aggregated data over the entire population. The results of ShesisPlus are quite variable between runs; some haplotypes are inferred in some runs but not in others. Overall, there is a rough agreement between the results of the ShesisPlus runs and the PolyHaplotyper results.

### Data set 2: simulated tetraploid population with 9 full-sib families

The simulated tetraploid population was composed of 9 FS families of 50 individuals. All FS families had one parent in common, so there were 10 parents and 450 FS individuals. As the correct genotypes were known from the simulation, the inferred haplotype genotypes of the individuals could be directly checked. We used PolyHaplotyper, SATlotyper and Happy-inf to analyze haploblocks of different sizes (3, 4, 5, 6 and 7 SNP markers). For each size 12 haploblocks were used. The results of all haploblocks are summarized in Table 3. In Additional file 2: Table S2 we show the results separately for haploblocks of different sizes.

**Table 3:** Haplotyping results of PolyHaplotyper, SATlotyper and Happy-inf with Data set 2

	PolyHaplotyper	SATlotyper	Happy-inf
Nr of haploblocks	60	60	60
Mean nr of haplotypes inferred	13.7	12.9	31.8
Mean true nr of haplotypes	13.9	13.9	13.9
Mean % haplotyped	89.6	100.0	100.0
Mean % correct (of all haplotyped)	98.7	42.4	33.4
Total nr correct	24,402	11,700	9220
Total nr incorrect	316	15,900	18,380
Total nr not haplotyped	2882	0	0
Run time (s)	5898	7024	1166

As in the Chrysanthemum data set, SATlotyper and Happy-inf haplotyped all fully genotyped individuals. In the simulated data set, with no missing marker data and no dosage errors, PolyHaplotyper haplotyped 89.6% of the individuals, which is more than the 74.9% haplotyped in the experimental data set. Another parallel between the two data sets is that the percentage individuals haplotyped correctly by PolyHaplotyper is very high (99.9% non-conflicting with parents in the Chrysanthemum data set, 98.7% correct in the simulated data), while these percentages were much lower with SATlotyper (81.7% and 42.4%) and with Happy-inf (86.2% and 33.4%, respectively). While for PolyHaplotyper the percentage of correct haplotypes was very high for all the haploblock sizes, an opposite trend was observed for SATlotyper (performing better in long haploblocks) and Happy-inf (performing better in short haploblocks) (Additional file 2: Table S2). For PolyHaplotyper there appears to be an upward trend in the percentage haplotyped and the percentage correctly haplotyped individuals, from haploblocks of 3 up to 6 SNP markers, but the results with 7 markers are worse. In the haploblocks with 7 markers 27 of the 12*9 = 108 FS families were treated as unrelated material because there were more than 150,000 possible parental haplotype combinations (150,000 being the default threshold), while this was the case for none of the FS families in haploblocks of 3, 4, and 5 markers and for only 2 FS families in haploblocks of 6 markers (results not shown).

Also for Dataset 2 we checked the effect of ignoring the FS families in PolyHaplotyper. Over the 60 haploblocks, without FS information only 37.3% of the individuals were haplotyped, versus 89.6% with FS information, and only 38.0% (versus 98.7%) of these were haplotyped correctly.

### Data set 3: simulated tetraploid population with 2 full-sib families and other material

The simulated tetraploid population was composed of 2 FS families of 50 individuals, sharing one parent, and 100 other individuals from the same random mating populations as the FS parents; in all there were 3 parents, 100 FS individuals and 100 other individuals. As in Dataset 2 the correct genotypes were known from the simulation, so the inferred haplotype genotypes of the individuals could be directly checked. We used PolyHaplotyper, SATlotyper and Happy-inf to analyze haploblocks of different sizes (3, 4, 5, 6 and 7 SNP markers). For each size 12 haploblocks were used. The results of all haploblocks are summarized in Table 4. In Additional file 2: Table S3 we show the results separately for haploblocks of different sizes and in Additional file 2: Table S4 for the different categories of material (FS individuals, FS parents, other material).

**Table 4:** Haplotyping results of PolyHaplotyper, SATlotyper and Happy-inf with Data set 3

	PolyHaplotyper	SATlotyper	Happy-inf
Nr of haploblocks	60	60	60
Mean nr of haplotypes inferred	16.0	13.9	33.3
Mean true nr of haplotypes	16.2	16.2	16.2
Mean % haplotyped	76.4	100.0	100.0
Mean % correct (of all haplotyped)	87.3	51.5	32.0
Total nr correct	8127	6272	3893
Total nr incorrect	1181	5908	8287
Total nr not haplotyped	2872	0	0
Run time (s)	1662	2095	527

Again SATlotyper and Happy-inf haplotyped all individuals, while PolyHaplotyper haplotyped less individuals than in Data set 2 (76.4% vs. 89.6%). The percentage individuals haplotyped correctly by PolyHaplotyper (87.3%) is again much higher that with SATlotyper (51.5%) and Happy-inf (32.0%), but lower than in Data set 2 (98.7%). The results of PolyHaplotyper were very good for the FS individuals and FS parents, but less good for the other (non-FS) material: only 59% of this was haplotyped on average, with 71.1% haplotyped correctly (Additional file 2: Table S4). The PolyHaplotyper results were somewhat worse for haplotypes of 7 markers than for the smaller haploblocks. Among the haploblocks with 7 markers 9 of the 12*2 = 24 FS families were treated as unrelated material because there were more than 150,000 possible parental haplotype combinations, while this was not the case in any of the smaller haploblocks (results not shown). Apart from this effect in haploblocks of 7 markers, the percentage of individuals correctly haplotyped by PolyHaplotyper and SATlotyper was not clearly affected by block size, but Happy-inf performed much better in small than in larger haploblocks (Additional file 2: Table S3).

Without using FS information only 44.4% of the individuals were haplotyped, versus 76.4% with FS information, and only 56.8% (versus 87.3%) of these were haplotyped correctly.

## Discussion

In this paper we consider short-range haplotyping. This is different from long-range or full chromosome haplotyping, where the aim is to characterize the different homologues in specific individuals. Such full-length haplotyping or phasing is often the result or by-product of linkage mapping, whereas our short-range haplotyping aims to generate multi-allelic data that can be used in downstream genetic analyses including linkage mapping. Some examples of software that can perform long-range haplotyping in polyploids are TetraploidSNPmap [22], TetraOrigin [23] and polymapR [24].

We describe a new approach for short-range haplotyping in polyploids, based on dosage data of bi-allelic markers, such as obtained from SNP genotyping arrays. In principle, SNP dosages could be obtained from sequence data as well, although current sequencing technologies result in less accurate dosage estimation compared to SNP arrays. This approach is implemented in the R package PolyHaplotyper. Although several methods and software packages have been published that are able to do this, our method is the first and so far the only that makes use of the presence of full-sib families (possibly linked through common parents). The use of this extra information results in a far lower rate of incorrectly haplotyped individuals, compared with two other packages (SATlotyper [13] and Happy-inf [19]) that do not use this information, and also compared to the results of PolyHaplotyper when the full-sib families are not specified. The low error rates make the haplotyping results of our method suitable for genetic analyses such as QTL analyses and linkage mapping, provided that the populations include one or more full-sib populations.

PolyHaplotyper is restricted to relatively small haploblocks: in practice the maxima are 8 markers in tetraploids and 6 markers in hexaploids. This theoretically allows to distinguish many different haplotypes, precisely 256 for 8 markers and 64 for 6 markers. However, depending on the variability in the population it may not always be possible to uniquely tag all different alleles of a gene. Other software, like Happy-inf which can deal with much larger haploblocks, is more suitable for that.

A possible future extension of PolyHaplotyper may involve a “smart” selection of bi-allelic markers to combine in a haploblock for the most informative result. A simple example is to avoid multiple markers that have the same dosages over the whole population, but more elaborate strategies are conceivable.

For PolyHaplotyper a slight upward trend appears in the percentage haplotyped and the percentage correctly haplotyped individuals with increasing numbers of markers per haploblock (Table 1, Additional file 2: Table S2A, although not in Additional file 2: Table S3A), but with 7 markers per haploblock the results become worse (although still better than those of SATlotyper and Happy-inf). Most likely this is due to the fact that with 7 markers per haploblock, many FS families were analyzed as unrelated material because the number of parental haplotype combinations exceeded the default threshold of 150,000. This could be improved by raising the threshold, but at a cost in computation time.

Another difference between PolyHaplotyper and the other packages to which we compared it, is the number of non-haplotyped individuals. SATlotyper haplotypes all individuals, including those where some or even all marker dosages are missing; Happy-inf haplotypes all fully genotyped individuals. In contrast, PolyHaplotyper only haplotypes the individuals where one haplotype combination is much more likely to be correct than all others. This may introduce a bias: for a given haploblock it may happen that some individuals are haplotyped and other are not, depending on their SNP dosage combination.

Further, individuals with some missing marker dosages are haplotyped by PolyHaplotyper if they are members of a full-sib family and there is only one possible haplotype combination, given the haplotyped parents, that matches the non-missing marker dosages.

This design choice explains part of the difference in the fraction of incorrectly genotyped individuals between PolyHaplotyper and the other packages. If individuals are haplotyped where multiple solutions are more or less equally likely, this results in less missing data but more incorrectly haplotyped individuals.

The enormous number of possible haplotyping solutions means that not all possibilities can be checked. In contrast to other methods, PolyHaplotyper approaches this problem as a puzzle with decisions at several stages involving different thresholds, aiming to prioritize the more likely solutions, rather than as a mathematical optimization in some high-dimensional landscape.

For efficient operation, PolyHaplotyper needs pre-calculated lists that contain all possible haplotype combinations, given the ploidy and the marker dosages. These tables take some time to compute, but once they are available they can be re-used for all analyses. For that reason, the time needed to compute these lists is not included in the run times mentioned earlier.

## Conclusion

PolyHaplotyper is an addition to the suite of polyploid haplotyping software. Like other software it has its own niche in which it performs well. For PolyHaplotyper, this niche involves the analysis of SNP array data to produce multi-allelic markers for use in downstream genetic analysis, in populations that include one or more full-sib families.

## Methods

### PolyHaplotyper algorithm

#### Population structure

In principle, populations with any genetic structure can be haplotyped by our method. However, the algorithm takes advantage of the presence of full-sib (FS) families and their parents, where groups of FS families sharing common parents are jointly taken into account. FS families include any progenies of crosses between two parental plants and as such include F_2_ progenies derived from the selfing of one F_1_ plant, backcross progenies derived from the cross of one F_1_ plant and one recurrent parent plant, etc. All individuals not belonging to one of the specified FS families or their parents are considered to be unrelated. All individuals must be of the same, even ploidy level.

#### Input data

The input includes the population structure, the haploblock compositions and the biallelic marker dosage data. The population structure indicates parents and progeny belonging to each FS family. The same individual may be the parent of more than one FS family. All other individuals occurring in the marker data are haplotyped as if they were unrelated, even if a pedigree is available. The haploblock composition describes which biallelic markers belong to each haploblock. The marker data consist of a matrix that for each biallelic marker gives the dosages of one of its alleles for all individuals; missing data are allowed.

We use the symbols *nmrk* for the number of bi-allelic markers (SNPs) in the haploblock and *nhap* for the total number of possible haplotypes, where *nhap* = 2^*nmrk*^. A (phasing) “solution” for an individual is a combination of haplotypes that fits (results in) the observed SNP marker dosages. Finally *Gmrk* is the genotype of an individual expressed as the allele dosages of all markers in the haploblock (where the dosage of each marker is in 0—ploidy), and *Ghap* is the genotype of an individual expressed as the dosages of all haplotypes (these dosages sum to ploidy). *Gmrk* are the observed SNP marker dosages and are the input of the haplotyping process; *Ghap* are the inferred haplotype combinations, the result of the haplotyping.

The main function, inferHaplotypes, is essentially a wrapper that performs the haplotyping for each haploblock separately; linkage between haploblocks is not taken into account. In short, the algorithm applied to each haploblock (in function hapOneBlock) consists of three stages:

- Stage 1: an inventory is made of haplotypes that are very likely present in the population, based on necessity (e.g. homozygous individuals) and a parsimony criterion
- Stage 2: full-sib families and their parents are haplotyped based on observed segregation ratios; any new inferred haplotypes are added to the inventory of haplotypes; FS families that cannot be haplotyped as such are re-defined as unrelated material
- Stage 3: the unrelated material (including any FS families added in stage 2) are haplotyped based on the inventory of known haplotypes and a parsimony criterion

These stages are elaborated below.

#### Stage 1: inventory of likely haplotypes

In this initial step, implemented in function inferHaps_noFS, we attempt to find a minimal set of haplotypes that together explain a large subset of the observed bi-allelic marker dosages. We apply an algorithm inspired by that of [25], developed for diploid populations, which aims to identify a parsimonious set of haplotypes. When there is a priori information available about haplotypes that are likely to be present in the population, this information is used.

This stage results in both a set of haplotypes known with some certainty to be present, and *Ghap* for many of the individuals. However, the purpose of this stage is only to obtain a set of haplotypes as input for the analysis of FS families (Stage 2) and the *Ghap* are ignored. If no FS families are present Stage 1 and 2 are skipped.

- Initially we identify haplotypes that are certain to be present in some minimum number of individuals. Individuals where all markers in the haploblock have a dosage equal to 0 or ploidy must be homozygous for a haplotype. Similarly, if only one of the markers has a dosage different from 0 or ploidy, two haplotypes are certain to be present. This can be generalized: if all combinations of haplotypes that explain the marker dosages of an individual contain a certain haplotype, that haplotype is known to be present. The use of a minimum threshold for the number of individuals (by default: 10%) reduces the chances to infer the presence of a haplotype due to dosage scoring errors.
  This step results in a number of confirmed haplotypes, that are added to a priori known haplotypes (if provided).
- Next, for each individual that cannot be completely explained by the already known haplotypes, we find the solutions that involve the least number of additional haplotypes, and we determine which haplotypes are required in all of these solutions. Haplotypes that are required in some minimum number of individuals (also by default 10%), together with haplotypes defined in step a, become the new set of known haplotypes.

We repeat step b until the set of known haplotypes does not change anymore. It may happen that the repetitions of this step do not converge to a single set of known haplotypes but cycle through a few different sets. In that case we use the results of the first execution of this step, which are based on the haplotypes identified with (almost) complete certainty in step a.

#### Stage 2: haplotyping of full-sib families

If FS families are specified, perhaps along with unrelated material, we first group the FS families into groups where the families are linked through shared parents. For each group we find an optimal solution, using the procedure described below. All haplotypes present in the parents according to the optimal solutions for all groups are assumed to be confirmed and are used as input for the haplotyping of the unrelated material (Stage 3). Stage 2 is summarized in Additional file 3: Fig. S1.

For each FS family we rely on the parental marker dosages to be non-missing and correct. If one or both parents have missing dosages for one or more markers in the haploblock, then for this haploblock the FS family and its parents are considered to be unrelated material. This is also the case for FS families where no acceptable solution is found, as will usually be the case if there are errors in the parental genotypes.

For FS families where both parents have full marker dosage information we apply function solveOneFS: consider the possible combinations of parental *Ghap*, each of which leads to a prediction of the haplotype segregation in the FS progeny. In calculating the expected haplotype segregation a specified frequency of double reduction is taken into account (default 2.5%). We convert the expected haplotype segregation into an expected segregation of marker allele dosage genotypes, taking into account a small probability of errors in dosage scores (default: 2.5%). We test the observed *Gmrk* segregation in the FS against this expected segregation, using a chi-squared test. In order to limit the bias of this test caused by categories with small number of expected individuals we group all expected *Gmrk* with less than 1 expected individual, together with the observed but unexpected *Gmrk*, in a single group. After this step we select the best parental *Ghap* combinations: those whose chi-squared *P* value is not less than 0.001 * the *P* value of the best combination and larger than some minimum threshold (default 10^–8^).

Depending on the *Gmrk* of the parents there may be a large number of possible parental *Ghap* combinations. Checking one parental *Ghap* combination takes appreciable time (a standard desktop computer with Intel i5 processor at 1.60 GHz, Windows10 can check about 200,000 parental *Ghap* combinations per hour). Therefore we try to consider the most likely parental *Ghap* first, which are the ones that involve only the haplotypes determined to be present after Stage 1. If no good solution is obtained with these selected haplotype combinations we consider all other possible *Ghap* as well, although it is possible to specify a maximum number of parental *Ghap* combinations (default: 150,000), above which the FS family will be treated as unrelated material.

Once all FS families in a group with shared parents have been analyzed, some of the FS families may have more than one possible solution and/or the solutions of FS families with a shared parent may be incompatible (i.e. each specifying a different *Ghap* for the shared parent). The best solution over the group is selected in function resolveGroupConflicts as follows. First we get rid of the incompatible solutions by iterating the following steps.

- For each parent in the group of linked FS families we consider all *Ghap* that are a potential solution for any of the FS families of which it is a parent. For each of these *Ghap* we determine for which of the FS families it is NOT a solution and we sum the numbers of individuals in these FSs. In this way for each potential *Ghap* of each parent in the group we know how many FS individuals in the group are not explained by it.
- If no such *Ghap* have unexplained FS families, all remaining FS families in the group are compatible and we stop this process. Else we remove the parental *Ghap* with most incompatible FS progeny from the set of possible solutions of each of the FS families of which this parent is a parent. If this was the last remaining solution of an FS family, the entire FS family is considered as incompatible and is removed from the group. All FS progeny in that population and its other parent (if not shared by other FS families in the group) are removed from the group and the process is repeated. The removed FS family and parent will be treated as unrelated material for this haploblock.

After this iteration there are one or more sets of *Ghap* for all parents that together are solutions for the entire (remaining) group. If more than one such set exists, we select the optimal one. The optimal solution is found by multiplying the chi-squared *P* values over all (remaining) FS families in the group and selecting the one(s) with the maximum combined *P* value. If multiple overall solutions are equally optimal, we assign *Ghap* to all parents and FS individuals that have the same, unique *Ghap* under all these solutions and leave the others unassigned.

For FS families where a single solution is found all possible marker genotypes (*Gmrk*) are known. For FS individuals with one or more missing marker dosages these are imputed (function imputeFSindiv) if the remaining markers allow only one of these possible *Gmrk*. In order to avoid over-interpretation, the imputed marker genotypes are accepted only if less than half of the FS family is imputed, and if the chi-squared fit including the imputed progeny is not too much worse than without these individuals (the chi-squared *P* value with imputation must be > 0.1 * the *P* value without imputation).

#### Stage 3: haplotyping of remaining material

After all groups of FS families have been considered we may be left with FS families (and parents) for which no solution was found or that had a solution incompatible with the other FS families in their group. These individuals, together with any material that was not part of a FS family, are jointly analyzed as a group of unrelated material. This is also the case if no FS families were defined in the original population.

In this analysis all haplotypes present in the now haplotyped FS families and any haplotypes specified as known are taken as known haplotypes. The initial analysis of this material in stage 3 is identical to stage 1 and also performed by function inferHaps_noFS. After this analysis some of the selected *Ghap* may contain haplotypes that are not in the set of known haplotypes because they occur in less than the minimum number of individuals. If so, we run one extra cycle of step b (described under stage 1) with a lower threshold (by default 1%, but at least 2 individuals, instead of 10%) for the number of individuals in which a haplotype should occur to be considered “known”. If this results in more individuals with a unique solution and if no individuals that had a unique solution now don’t have one anymore, then we accept the result of this final cycle.

### Implementation

The algorithm described above is implemented in an R package [26] named PolyHaplotyper which is available from CRAN [27]. The entire haplotyping process over multiple haploblocks in a single population (which may consist of multiple FS families, their parents and unrelated material) is performed through a single call to function inferHaplotypes. Apart from this function the package provides functions that help to format the data set, to merge replicate samples from the same individuals and to produce overviews and statistics.

Apart from the relations between FS families and their (possibly shared) parents, the haplotyping process does not make use of known pedigree relations. However, if a pedigree is available, PolyHaplotyper allows to check for each parents-offspring trio (or pair, if only one parent is available) whether the inferred haplotype composition of the offspring is compatible with that of the parents.

An important characteristic of our method is that it relies on considering all possible combinations of haplotypes that yield the observed marker dosages. The number of haplotype combinations quickly becomes astronomical with increasing ploidy level and increasing number of markers per haploblock. The implementation allows to generate the combinations as required, but the computation time then quickly becomes prohibitive. It is much more efficient to tabulate all haplotype combinations for all marker dosage combinations in advance, so that the table for the desired ploidy can be loaded at the start of a run. The package also provides functions to calculate these tables. In practice, these pre-calculated tables are currently limited to 8 markers per haploblock for tetraploids and 6 for hexaploids. Larger numbers would require a different access method as it would become impossible to store these tables in memory and the indices to the tables would overrun the capacity of the 32-bit integers in R.

### Comparisons with other software

Our software is not the first that is aimed at short-range haplotyping in polyploids based on SNP dosage data, but to our knowledge it is the first that makes use of known FS families in the data. We compared the results of our software with those of SATlotyper [13] downloaded from [28], Happy-inf [19] downloaded from [29] and SHEsisPlus [17, 18] accessed online at [21]. All were used with their recommended or default settings. Functions are supplied in the PolyHaplotyper package to convert PolyHaplotyper-formatted input data to the formats required by these packages, and to reformat their output to PolyHaplotyper output format. Further, PolyHaplotyper contains functions to convert marker data simulated with PedigreeSim (Voorrips and Maliepaard, 2012) to the input format of PolyHaplotyper, and to compare haplotyping results with “true” simulated genotypes generated by PedigreeSim.

### Data sets

#### Data set 1: hexaploid Chrysanthemum population

Data set 1 contains SNP dosage data obtained from chrysanthemum using a dedicated Axiom SNP array [20]. The hexaploid population consists of four FS families of 405, 53, 76 and 37 individuals, with 7 parents (families 1 and 4 share a common parent) and 53 unrelated individuals. For this population the true genotypes are unknown, but a pedigree is available that allows to check for inconsistent haplotype assignments. The data set contains 189 SNP markers, grouped in 43 haploblocks of 4 or 5 SNPs. Markers grouped in a haploblock mapped on the same sequence contig, based on sequence data generated prior to the array development.

#### Data set 2: simulated tetraploid population with 9 FS families

This tetraploid data set is the result of a simulation performed using PedigreeSim [30]. The population consists of 9 FS families of 50 individuals each, all sharing one common parent. The parents have been simulated so that they are genetically structured into three groups of related individuals. The number of founder alleles segregating per locus ranges between 14 and 26. However, the number of haploblock alleles is lower, due to the limited information content of small-size haploblocks. SNP-based heterozygosity per individual (i.e. the percentage of heterozygous calls over the total number of SNPs per individual) ranges from 73 to 85%. Results are presented for 300 biallelic (SNP) markers grouped in 60 haploblocks of 3—7 markers; all markers in a haploblock were simulated at the same genetic map position, i.e. no recombination could occur within haploblocks. The haplotyping results were compared to the true haplotype compositions, which are known from the simulation results.

#### Data set 3: simulated tetraploid population with 2 FS families and other material

This tetraploid data set is the result of a simulation performed using PedigreeSim [30]. The population consists of 2 FS families of 50 individuals each, sharing one common parent. The parents have been simulated as originating from two distinct random-mating populations. In addition there are two groups of 50 individuals, one group belonging to each of these two random-mating populations. Results are presented for 300 biallelic (SNP) markers grouped in 60 haploblocks of 3–7 markers; all markers in a haploblock were simulated at the same genetic map position, i.e. no recombination could occur within haploblocks. The number of haplotypes per haploblock ranges from 7 to 29, with an average of 16.2. The haplotyping results were compared to the true haplotype compositions, which are known from the simulation results.

## Supplementary Information

**Additional file 1.** A basic explanation of the concepts of haploblocks and haplotyping, as used in this article.

**Additional file 2. Table S1.** Total haplotype counts in three runs of ShesisPlus and of PolyHaplotyper, for four haploblocks of Data set 1. **Table S2.** Haplotyping results with Data set 2, separated by haploblock size. **Table S3.** Haplotyping results with Data set 3, separated by haploblock size. **Table S4.** Haplotyping results with Data set 3, separated by material.

**Additional file 3. Fig. S1.** Stage 2 of the haplotyping process.

**Additional file 4.** Dataset1_chrysanthemum.RData: A file containing Data set 1 in a format that can be loaded in R.

**Additional file 5.** Dataset1_script.R: An R script illustrating how Data set 1 was processed with PolyHaplotyper, and how the comparisons with SATlotyper, Happy-inf and SHEsisPlus were made.

**Additional file 6.** Dataset2_script.R: An R script illustrating how Data set 2 was processed with PolyHaplotyper, and how the comparisons with SATlotyper and Happy_inf were made.

**Additional file 7.** Dataset2_sim_9_FS_families.RData: A file containing Data set 2 in a format that can be loaded in R.

**Additional file 8.** Dataset3_script.R: An R script illustrating how Data set 3 was processed with PolyHaplotyper, and how the comparisons with SATlotyper and Happy_inf were made.

**Additional file 9.** Dataset3_sim_2_FS_and_other.RData: A file containing Data set 3 in a format that can be loaded in R.

## Data Availability

The data sets used in the current study, and R scripts detailing how they were analyzed are included in this article as Additional files 1, 2, 3, 4, 5, 6, 7, 8 and 9. The algorithm described in this article is implemented in an R package named PolyHaplotyper which is publicly available from CRAN [27] at https://cran.r-project.org/web/packages/PolyHaplotyper/.

## Abbreviations

CRAN: The Comprehensive R Archive Network
FS: Full-sib
GWAS: Genome-wide association study
QTL: Quantitative trait locus
SNP: Single-nucleotide polymorphism
SSR: Short sequence repeat
