# Supplementary material for: PolyHaplotyper: haplotyping in polyploids based on bi-allelic marker dosage data
Source: BMC Bioinformatics. 2022 Oct 23;23:442. doi: 10.1186/s12859-022-04989-0 (PMC9590153; doi:10.1186/s12859-022-04989-0)
Supplement: Supplementary file 1 — Additional file 1. A basic explanation of the concepts of haploblocks and haplotyping, as used in this article. [file 12859_2022_4989_MOESM1_ESM.pdf]

Haploblocks and haplotyping

We define a haploblock as a genomic region containing several SNP markers, with (almost) no recombination over the population studied. For example a haploblock could consist of a single contig, but also of a set of SNPs between which no recombination is observed in a mapping population; or a set of SNPs known to be located within a few thousand base pairs according to a reference sequence. The SNPs we consider are bi-allelic: each SNP has only 2 alleles (SNPs with 3 or 4 alleles do occur but we don't use them in our approach).

The haplotyping procedure implemented in PolyHaplotyper is based on SNP dosage data: for each separate SNP the allele dosage of the individuals in the population is known. This information is typically obtained from SNP arrays. For each SNP and each sample two signal intensities are produced, and from these signals the allele dosage is inferred, using software such as fitPoly (Voorrips et al, 2011; Zych et al, 2019). The allele dosage is expressed for one of the two alleles of each SNP, typically the alternative allele (as opposed to the reference) allele, if a reference sequence is available. The allele dosage is an integer number, ranging from 0 to the ploidy level of the individual. PolyHaplotyper is developed especially for polyploid populations although it will also work with diploid populations.

In the following example we have a haploblock that is composed of 3 SNPs, names SNP1, SNP2 and SNP3. With 3 SNPs, 8 different haplotypes can be distinguished (Table1; the reference allele of each SNP is shown as 0, the alternative allele as 1):

Table 1. The 8 possible haplotypes at a haploblock containing 3 SNPs.

	haplotype							
	a	b	c	d	e	f	g	h
SNP1	0	0	0	0	1	1	1	1
SNP2	0	0	1	1	0	0	1	1
SNP3	0	1	0	1	0	1	0	1

We will consider the haploblock as a genetic locus, and the 8 haplotypes as the possible alleles at that locus. The genotype of a tetraploid individual at this haploblock can be expressed as a combination of 4 alleles (haplotypes). It could be homozygous (e.g. bbbb), it could have 4 different alleles (e.g. abef) or have other combinations of alleles such as bbee, accc, addf etc.

The genotype of the individuals in terms of haplotypes is not directly observed. Instead, we observe the allele dosages at the 3 SNPs separately. For example, we observe dosage 2 at SNP1, 0 at SNP2 and 1 at SNP3. Now there are multiple possible combinations of haplotypes that are compatible with these SNP dosages (Table 2).

Table 2. Two possible haploblock genotypes, both compatible with observed dosages 2, 0, 1 at SNP1, SNP2 and SNP3 respectively.

	Genotype aaef					Genotype abee				
	a	a	e	f	SNP dosage	a	b	e	e	SNP dosage
SNP1	0	0	1	1	2	0	0	1	1	2
SNP2	0	0	0	0	0	0	0	0	0	0
SNP3	0	0	0	1	1	0	1	0	0	1

Haplotyping is the process of determining the correct combination of haplotypes at a haploblock. As we saw in Table 2, for a given set of observed SNP dosages multiple combinations of haplotypes may be possible. Given just the SNP dosages of one single individual, it is not possible to determine which

genotype is correct. However given a population of individuals it may be possible to haplotype them all, or at least part of them, assuming that recombination within the haploblock occurs so little that it can be ignored.

A widely used criterion is that the number of haplotypes needed to explain all individuals should be minimized. If a pedigree is known we can also impose restrictions based on Mendelian inheritance: the haplotypes in an individual must also be present in its parents, and the segregation ratios in a full-sib family should match the genotypes of the parents. In PolyHaplotyper all these criteria are applied.
