# Supplementary material for: PolyHaplotyper: haplotyping in polyploids based on bi-allelic marker dosage data
Source: BMC Bioinformatics. 2022 Oct 23;23:442. doi: 10.1186/s12859-022-04989-0 (PMC9590153; doi:10.1186/s12859-022-04989-0)
Supplement: Supplementary file 2 — Additional file 2. Table S1. Total haplotype counts in three runs of ShesisPlus and of PolyHaplotyper, for four haploblocks of Data set 1. Table S2. Haplotyping results with Data set 2, separated by haploblock size. Table S3. Haplotyping results with Data set 3, separated by haploblock size. Table S4. Haplotyping results with Data set 3, separated by material. [file 12859_2022_4989_MOESM2_ESM.pdf]

Supplementary Table 1. Total haplotype counts in three runs of ShesisPlus and of PolyHaplotyper, for four haploblocks of Data set 1.

ctg001				
Haplotype[a]	SSP-1[b]	SSP-2	SSP-3	PolyHaplotyper
1111	0	0	0	79
1121	2672	2672	2672	2715
1221	0	0	0	1
1222	307	307	307	313
2121	510	510	510	522
total	3489	3489	3489	3630
ctg002				
Haplotype	SSP-1	SSP-2	SSP-3	PolyHaplotyper
1222	0	0	0	31
2122	321	321	321	320
2211	193	193	0	193
2212	487	487	680	477
2221	0	0	194	0
2222	2568	2568	2375	2555
total	3569	3569	3570	3576
ctg003				
Haplotype	SSP-1	SSP-2	SSP-3	PolyHaplotyper
11112	0	117	0	116
21112	137	137	137	134
21121	151	107	218	211
21122	2469	2308	2418	2169
21221	0	111	0	0
21222	0	200	145	307
22122	111	284	111	279
22222	118	0	118	0
total	2986	3264	3147	3216
ctg004				
Haplotype	SSP-1	SSP-2	SSP-3	PolyHaplotyper
11112	0	129	0	0
12112	198	120	145	234
12121	0	0	0	4
21112	204	206	335	322
21121	130	0	0	0
22112	2719	2666	2641	2520
22121	0	221	118	211
22122	0	0	0	2
22212	116	116	116	109
total	3367	3458	3355	3402

[a]: Haplotypes are represented with the alleles of the 4 or 5 SNPs in the haploblocks coded as 1 or 2, as in the output of ShesisPlus

[b]: SSP-1, SSP-2, SSP-3: three runs of ShesisPlus

Supplementary Table 2. Haplotyping results with Data set 2, separated by haploblock size..

A. PolyHaplotyper

SNP markers per haploblock	3	4	5	6	7	all
Nr of haploblocks	12	12	12	12	12	60
Mean nr of haplotypes inferred	7.6	11.1	15.1	17.2	17.7	13.7
Mean true nr of haplotypes	7.6	11.3	14.9	17.1	18.5	13.9
Mean % haplotyped	87.1	90.1	92.5	94.5	83.6	89.6
Mean % correct (of all haplotyped)	99.1	98.8	98.9	99.7	97.0	98.7
Total nr correct	4764	4911	5049	5200	4478	24402
Total nr incorrect	45	62	55	17	137	316
Total nr not haplotyped	711	547	416	303	905	2882

B. SATlotyper

SNP markers per haploblock	3	4	5	6	7	all
Nr of haploblocks	12	12	12	12	12	60
Mean nr of haplotypes inferred	7.3	10.5	13.2	15.5	18.2	12.9
Mean true nr of haplotypes	7.6	11.3	14.9	17.1	18.5	13.9
Mean % haplotyped	100.0	100.0	100.0	100.0	100.0	100.0
Mean % correct (of all haplotyped)	35.3	34.6	37.8	38.5	65.8	42.4
Total nr correct	1949	1911	2085	2124	3631	11700
Total nr incorrect	3571	3609	3435	3396	1889	15900
Total nr not haplotyped	0	0	0	0	0	0

C. Happy-inf

SNP markers per haploblock	3	4	5	6	7	all
Nr of haploblocks	12	12	12	12	12	60
Mean nr of haplotypes inferred	7.8	14.5	25.8	42.7	68.5	31.8
Mean true nr of haplotypes	7.6	11.3	14.9	17.1	18.5	13.9
Mean % haplotyped	100.0	100.0	100.0	100.0	100.0	100.0
Mean % correct (of all haplotyped)	61.0	36.5	33.3	23.3	13.0	33.4
Total nr correct	3369	2013	1836	1287	715	9220
Total nr incorrect	2151	3507	3684	4233	4805	18380
Total nr not haplotyped	0	0	0	0	0	0

Supplementary Table 3. Haplotyping results with Data set 3, separated by haploblock size.

A. PolyHaplotyper

SNP markers per haploblock	3	4	5	6	7	all
Nr of haploblocks	12	12	12	12	12	60
Mean nr of haplotypes inferred	7.2	10.3	14.9	18.4	29.3	16.0
Mean true nr of haplotypes	7.5	12.1	17.0	20.8	23.7	16.2
Mean % haplotyped	85.7	76.6	81.6	76.1	62.1	76.4
Mean % correct (of all haplotyped)	92.5	89.8	88.4	88.6	74.2	87.3
Total nr correct	1931	1675	1757	1642	1122	8127
Total nr incorrect	156	191	231	212	391	1181
Total nr not haplotyped	349	570	448	582	923	2872

B. SATlotyper

SNP markers per haploblock	3	4	5	6	7	all
Nr of haploblocks	12	12	12	12	12	60
Mean nr of haplotypes inferred	7.0	10.3	14.2	17.4	20.8	13.9
Mean true nr of haplotypes	7.5	12.1	17.0	20.8	23.7	16.2
Mean % haplotyped	100.0	100.0	100.0	100.0	100.0	100.0
Mean % correct (of all haplotyped)	57.0	45.4	42.5	51.7	60.8	51.5
Total nr correct	1388	1107	1036	1260	1481	6272
Total nr incorrect	1048	1329	1400	1176	955	5908
Total nr not haplotyped	0	0	0	0	0	0

C. Happy-inf

SNP markers per haploblock	3	4	5	6	7	all
Nr of haploblocks	12	12	12	12	12	60
Mean nr of haplotypes inferred	7.9	14.5	27.0	45.3	71.7	33.3
Mean true nr of haplotypes	7.5	12.1	17.0	20.8	23.7	16.2
Mean % haplotyped	100.0	100.0	100.0	100.0	100.0	100.0
Mean % correct (of all haplotyped)	60.6	44.1	26.8	17.4	10.9	32.0
Total nr correct	1475	1075	654	423	266	3893
Total nr incorrect	961	1361	1782	2013	2170	8287
Total nr not haplotyped	0	0	0	0	0	0

Supplementary Table 4. Haplotyping results with Data set 3, separated by material.

	PolyHaplotyper	SATlotyper	Happy-inf
Fullsib individuals (100)			
mean % haplotyped	93.4	100.0	100.0
Mean % correct (of all haplotyped)	97.2	51.4	35.6
Fullsib parents (3)			
mean % haplotyped	92.8	100.0	100.0
Mean % correct (of all haplotyped)	100.0	53.3	33.9
Other individuals (100)			
mean % haplotyped	59.0	100.0	100.0
Mean % correct (of all haplotyped)	71.1	51.5	28.2
All individuals (203)			
mean % haplotyped	76.4	100.0	100.0
Mean % correct (of all haplotyped)	87.3	51.5	32.0
