# Supplementary material for: PolyHaplotyper: haplotyping in polyploids based on bi-allelic marker dosage data
Source: BMC Bioinformatics. 2022 Oct 23;23:442. doi: 10.1186/s12859-022-04989-0 (PMC9590153; doi:10.1186/s12859-022-04989-0)
Supplement: Supplementary file 3 — Additional file 3. Fig. S1. Stage 2 of the haplotyping process. [file 12859_2022_4989_MOESM3_ESM.pdf]

Group connected FS families
(FS families linked by common parents)

For each group of FS families:

For each FS in group separately:

- Get all parental haplotype combinations
- Check each combination against segregation in FS
- Select best parental combinations

Select parental haplotype combinations that fit all FS in group simultaneously
(removing non-matching FS from group if needed)

Select the set of parental haplotype combinations that best fits the segregation in all FS in group

Assign a haplotype combination to all FS progeny in the group matching their marker dosages,
if one possible combination of parental haplotypes is much more likely than all others

Add all parental haplotypes from all FS groups to the inventory of haplotypes, for haplotyping of non-FS samples

Supplementary figure 1: Stage 2 of the haplotyping process
